# Molecularly Characterized Solvent Extracts and Saponins from *Polygonum hydropiper* L. Show High Anti-Angiogenic, Anti-Tumor, Brine Shrimp, and Fibroblast NIH/3T3 Cell Line Cytotoxicity

**DOI:** 10.3389/fphar.2016.00074

**Published:** 2016-03-31

**Authors:** Muhammad Ayaz, Muhammad Junaid, Farhat Ullah, Abdul Sadiq, Fazal Subhan, Mir Azam Khan, Waqar Ahmad, Gowhar Ali, Muhammad Imran, Sajjad Ahmad

**Affiliations:** ^1^Department of Pharmacy, University of MalakandKhyber Pakhtoonkhwa, Pakistan; ^2^Department of Pharmacy, University of PeshawarPeshawar, Pakistan; ^3^H.E. J. Research Institute of Chemistry, International Center for Chemical and Biological Sciences, University of KarachiKarachi, Pakistan

**Keywords:** *Polygonum hydropiper*, saponin, anti-angiogenic, chorioallantoic membrane assay, fibroblast cell line, MTT assay

## Abstract

*Polygonum hydropiper* is used as anti-cancer and anti-rheumatic agent in folk medicine. This study was designed to investigate the anti-angiogenic, anti-tumor, and cytotoxic potentials of different solvent extracts and isolated saponins. Samples were analyzed using GC, Gas Chromatography–Mass Spectrometry (GC–MS) to identify major and bioactive compounds. Quantitation of antiangiogenesis for the plant's samples including methanolic extract (Ph.Cr), its subsequent fractions; *n*-hexane (Ph.Hex), chloroform (Ph.Chf), ethyl acetate (Ph.EtAc), *n*-Butanol (Ph.Bt), aqueous (Ph.Aq), saponins (Ph.Sp) were performed using the chick embryo chorioallantoic membrane (CAM) assay. Potato disc anti-tumor assay was performed on *Agrobacterium tumefaciens* containing tumor inducing plasmid. Cytotoxicity was performed against *Artemia salina* and mouse embryonic fibroblast NIH/3T3 cell line following contact toxicity and MTT cells viability assays, respectively. The GC–MS analysis of Ph.Cr, Ph.Hex, Ph.Chf, Ph.Bt, and Ph.EtAc identified 126, 124, 153, 131, and 164 compounds, respectively. In anti-angiogenic assay, Ph.Chf, Ph.Sp, Ph.EtAc, and Ph.Cr exhibited highest activity with IC_50_ of 28.65, 19.21, 88.75, and 461.53 μg/ml, respectively. In anti-tumor assay, Ph.Sp, Ph.Chf, Ph.EtAc, and Ph.Cr were most potent with IC_50_ of 18.39, 73.81, 217.19, and 342.53 μg/ml, respectively. In MTT cells viability assay, Ph.Chf, Ph.EtAc, Ph.Sp were most active causing 79.00, 72.50, and 71.50% cytotoxicity, respectively, at 1000 μg/ml with the LD_50_ of 140, 160, and 175 μg/ml, respectively. In overall study, Ph.Chf and Ph.Sp have shown overwhelming results which signifies their potentials as sources of therapeutic agents against cancer.

## Introduction

Angiogenesis is the growth of new capillaries from pre-existing capillaries and post-capillary venules. It is a highly controlled process that usually occurs during wound healing, embryonic and corpus luteum development (Folkman, 1972). It is now well-established that many diseases are caused by persistent unregulated angiogenesis, like tumor growth is highly dependent on vascular growth. Those tumors which lack angiogenesis stay dormant and quick logarithmic growth follow the attainment of blood supply (Folkman and Klagsbrun, 1987). Tumors angiogenic control seems to be activated when angiogenic inhibitors and stimulators balance are shifted toward pro-angiogenic milieu (Hanahan and Folkman, 1996). Due to this reason, interest in the identification and development of anti-angiogenic drugs is tremendously increased. In contrast, induction of angiogenesis is also of great interest in treatment of ischemic diseases (Horvath et al., 1997). Administration of angiogenic growth factors in the form of recombinant protein or by gene transfer to the ischemic animal models has shown to increase nutrient perfusion via neo-vascularization. Novel gene therapy technologies and advancement in animal modeling have enabled scientists to expand therapeutic angiogenesis strategies applied in animal models of myocardial ischemia and coronary artery diseases. In this regard, several potential drugs with angiogenic activity are under investigations (Silvestre and Levy, 2002).

The chick embryo chorioallantoic membrane (CAM) is a model frequently used to evaluate angiogenic and anti-angiogenic properties of potential drugs (Ribatti et al., 2001). Using this method, angiogenic response occurs in 72–96 h after stimulation in the form of increased blood vessels density around the implant, with the vessels radially converging toward the center (Ribatti et al., 1995). On the other hand, when an anti-angiogenic compound is applied, the vessels around the implant become less dense or even vanish completely (Vacca et al., 1999). Quantitative analysis of vessels in large amount of CAM models is used to screen drugs and samples of plants. Some traditional Chinese herbal drugs have been reported to be effective in the therapy of ischemic heart diseases and cancer (Wang et al., 2004).

Cancer is a life-threatening disease and is a serious health problem worldwide. It represents a group of diseases characterized by uncontrolled proliferation of abnormal cells which invade and disrupt nearby tissues (Gennari et al., 2007). Due to toxicity problems, high cost and adverse side effects associated with the use of synthetic anticancer drugs, natural products is the most potential alternative source of useful anticancer drugs. The isolation of novel anticancer drugs like vincristine and vinblastine from plant sources provide convincing evidence that plants are potential sources of novel anticancer drugs. Potato disc assay is rapid, economical, and reliable bioassay that provides basis for the possible anticancer and anti-tumor utility of test samples. The inhibition of *A. tumefaciens* induced tumors (or Crown Gall) in potato discs, is an assay based on antimitotic activity and can detect a broad range of anti-tumor effects (McLaughlin, 1991). The assay is based on the hypothesis that anti-tumor drugs might inhibit the growth of tumors both in plant and animals, since some tumorogenic mechanisms are quite related in plants and animals (McLaughlin and Rogers, 1998). Crown Gall tumor is a neoplastic illness in plants caused by *A. tumefaciens*. The bacterium contain Ti (tumor inducing)-plasmids that carry genetic information (T-DNA) which upon infection transforms normal or wounded plant cells into independent tumor cells (Coker et al., 2003). The Ti-plasmid causes the plants' cells to multiply rapidly without going through apoptosis, resulting in tumor development, like in nucleic acid content and histology to human and animal cancers (Binns and Thomashow, 1988). Similarly, brine shrimp cytotoxicity assay is an important scientific tool for the preliminary cytotoxic analysis of natural and synthetic drugs before more complex and advance studies. As far as the phytochemicals are concerned, they possess pronounced biological potentials i.e., flavonoids are well-known for antioxidant potential, saponins for cytotoxic potential, alkaloids for antimicrobial potential, and have been reported by numerous investigators. In fact these secondary metabolites confer pharmacological potential to plants.

*P. hydropiper* belong to *Polygonaceae*, a family having about 50 genera, 1200 species and is traditionally used to treat cancer (Hartwell, 1970), hypertension, and cardiovascular diseases (Qureshi et al., 2006). We previously reported antioxidant, anticholinesterase (Ayaz et al., 2014a, 2015), phytotoxic, and anthelmintic potentials of *P. hydropiper* (Ayaz et al., 2014b). Domestically, its decoction is used as diuretic, ant-rheumatic, anti-inflammatory, haemostatic, and to relieve toothache (Popovic et al., 2014). Other species of *Polygonaceae* family have been reported for anti-tumor potentials (Mazid et al., 2011; Ahmad et al., 2016) and effectiveness in cerebral ischemia (Chan et al., 2003), Parkinson's disease (Chen et al., 2007) and as neuroprotective (Li et al., 2005). Based on the ethnomedicinal uses and research work on the related species, this study was designed to investigate anti-angiogentic, anti-tumor, and cytotoxic potentials of *P. hydropiper* extracts, crude saponins, and narrow down the search for isolation of novel anticancer compounds from this valuable plant.

## Material and methods

### Chemicals and drugs

Etoposide (E2600000 Fluka) CAS 33419-42-0, vincristine sulfate (V8388 Sigma-Aldrich) CAS 2068-78-2, Dulbecco's Modified Eagle's medium (DMEM; Sigma), Fetal Bovine Serum (FCS) (Gibco), 3-(4,5-dimethylthiazol-2-yl)- 2,5-diphenyltetrazolium bromide (MTT; Sigma), Dexamethasone (GlaxoSmithKline, Pakistan), Dimethyl-Sulfoxide (DMSO; RCI Labscan, Bankok, Thialand) Soybean Casein Digest Agar (Oxoid Ltd, Basingstoke, Hampshire, England) medium. The solvents used were of analytical grade purchased from Sigma Aldrich Chemie (GmbH, Riedstrasse, Steinheim, Germany).

### Plant materials, extraction, and fractionation

*P. hydropiper* was collected from Talash Valley, District Dir (Lower) Khyber Pakhtoonkhwa Pakistan in July, 2013 and was authenticated by Dr. Gul Rahim Arid Agriculture University, Rawalpindi, Pakistan. The plant sample was deposited at the herbarium of University of Malakand, Chakdara (Dir), Pakistan with voucher (H.UOM.BG.107). Plant materials were washed with distilled water to remove dust and was shade dried for 30 days. Dried materials were coarsely crushed and the powdered material (4.5kg) was soaked in 80% methanol (22 L) in large container for 15 days with occasional shaking. Solvent extraction was done in triplicate, added to the original extract and filtered using muslin cloth and filter paper (Konan et al., 2008). The filtrate was concentrated using rotary evaporator (Heidolph Laborota 4000, Schwabach, Germany) under reduced pressure at 40°C which resulted in 290 g (6.44%) of dark brown semisolid mass. Crude methanolic extract (250 g) of *P. hydropiper* (Ph.Cr) was suspended in 500 ml of distilled water and consequently partitioned with *n*-hexane (3 × 500 ml), chloroform (3 × 500 ml), ethyl acetate (3 × 500 ml), *n*-butanol (3 × 500 ml), and water (3 × 500 ml). Finally, 68 g (27.2%) Ph.Hex, 27 g (10.8%) Ph.Chf, 13 g (5.2%) Ph.EtAc, 11 g (4.4%) Ph.Bt, and 37 g (14.8%) Ph.Aq were obtained.

### Extraction of saponins

Dried plant material (60 g) was soaked with 100 ml of 20% ethanol in a conical flask and was heated at 55°C for 4 h using water bath with occasional shaking. Subsequently, it was filtered and again extracted with 200 ml of 20% ethanol. Volume obtained was concentrated to 40 ml via water bath and transferred it to a separating funnel. Further, 20 ml of diethyl ether was added to the separating funnel and was shacked vigorously. Among the two layers formed (Diethyl ether and water), aqueous layer was utilized by adding 60 ml of *n*-butanol to it. The combined mixture (aqueous and n-butanol layer) was washed two times with 5% NaCl solution. Finally, it was concentrated using water bath to get saponins (9 g) with 15% yield (Khan et al., 2011).

### Gas chromatography–mass spectrometry (GC/MS) analysis

Samples were initially subjected to GC analysis using an Agilent USB-393752 gas chromatograph (Agilent Technologies, Palo Alto, CA, USA) with HHP-5MS 5% phenylmethylsiloxane capillary column (30 m × 0.25 mm × 0.25 μm film thickness; Restek, Bellefonte, PA) equipped with an FID detector. The initial temperature of the oven was retain at 70°C for 1 min, followed by increase at the rate of 6°C/min to 180°C for 5 min and lastly at the rate of 5°C/min to 280°C for 20 min. Injector and detector temperatures were set at 220°C and 290°C, respectively. Helium was used as a carrier gas with a flow rate of 1 ml/min, and diluted the plant samples (1/1000 in *n*-pentane, v/v) of 1.0 μl were injected manually in the split-less mode. GC/MS analysis of samples were processed using the same column and experimental conditions.

### Identification of components

Compounds were recognized by comparison of their retention times with those of authentic compounds in the literature. Further, identification were done via the spectral data obtained from the Wiley and NIST libraries, as well as comparisons of the fragmentation pattern of the mass spectra with data published in the literature (Stein et al., 2002; Adams, 2007).

### Chorioallantoic membrane (CAM) assay

Anti-angiogenic potential of plant extracts and saponins were determined by CAM assay (Nguyen et al., 1994). The fertilized domestic chicken eggs purchased from poultry trader Chakdara, Pakistan, were incubated for 3–4 days at 37°C in a humidified incubator (HYSC Korea (BI-81/150/250) and were slowly moved at least three times a day. After the completion of incubation period, the seven day old eggs were observed under flash light to identify and encircle the embryo head. Thereafter, a tiny hole was drilled at the narrow end of the eggs and 0.5–1 ml of albumin was aspirated using eighteen gauge hypodermic needle so that yolk sacs drop away from the shell membrane. The shell around the embryo air sac was detached via forceps and the shell membrane at the base of air sac was peel away. On 8th day, a thermanox cover slip loaded with different samples (10 μl) was carefully placed on the surface of CAM and were incubated. After 3 days, an appropriate amount of methanol and acetone mixture (1:1) was injected into the embryo chorioallantois using a 33 gauge needle. The CAM was cut out from eggs and the numbers of vessels were observed. Vessels radially converging in the direction of the center were counted under a microscope. At least twenty eggs were used for each sample dose. The % of increase and inhibition were calculated using formula;

$$\begin{array}{l} \text{Percent inhibition}=\\ \;\frac{\begin{array}{c} \text{No of vessel in CAM treated with normal saline}\\ -\text{No of vessel in CAM treated with plant samples} \end{array}}{\text{No of vessel in CAM treated with normal saline}}\times 100 \end{array}$$

### Potato disc anti-tumor bioassay

#### Preparation of plant extracts-*A. tumefaciens* mixture

The assay was performed according to the established procedure described by McLaughlin and Rogers (McLaughlin, 1998). *A. tumefaciens* (strain B6) containing Ti (tumor inducing)-plasmid was cultured on Soybean Casein Digest Agar (SCDA) overnight at 25°C. Different dilutions of plant extracts ranging from 31.25–1000 μg/ml were prepared in DMSO and were filtered. Inoculums containing five concentrations of the extracts (31.25, 62.50, 125, 250, 500, and 1000 μg/ml), *Agrobacterium* culture corresponding to 1 × 10^8^ CFU were prepared. Control solution was prepared by adding 50 μl of filtered DMSO to 450 μl of sterile distilled water, and then mixed with 500 μl *A. tumefaciens* broth culture.

#### Potato discs preparation

Red skinned potatoes were purchased from the local market near University of Malakand Chakdara, Pakistan. Using sterile cork borer, potato discs of 2 mm height and 8 mm diameter were made. These discs were surface sterilized with 1% HgCl_2_ solution for 4–5 min followed by washing with distilled water. These were allowed to dry aseptically for 20 min. The discs were placed on plates containing autoclaved agar medium (1.5%) using sterile forceps. Finally, the top surface of each potato disc was inoculated with 50 μl of plant extract-bacterium mixture. The plates were sealed with parafilm and incubated at 28°C in dark. After 15–20 days, potato discs were stained with Lugol's solution (10% KI + 5% I_2_) and tumors were counted under dissecting microscope. Vincristine sulfate and solvents system were taken as positive and negative control, respectively. Test was repeated three times and data was analyzed statistically.

### *In-vitro* anti agrobacterium assay

#### Disk diffusion assay

In order to check the effect of plants samples on the growth of Agrobacterium and hence on tumor formation, a qualitative to semi quantitative disc method was used following previously reported procedure (Bauer et al., 1966). Briefly, nutrient agar plates prepared aseptically were inoculated with test organisms under laminar flow hood. Sterile paper disks of 6 mm diameter (Whatman International, CAT: 2017-006) impregnated with different concentrations of extracts were placed equidistantly onto the surface of already inoculated Petri dishes using sterile forceps. Blank discs impregnated with DMSO/solvents were used as negative control whereas ceftriaxone discs (Geltis, Shaigan Pharmaceuticals) were used as positive control. The plates were incubated at 37°C for 24 h and zones of inhibition were measured around the bores.

#### Determination of minimum inhibitory concentrations (MICs)

For determination of MICs, nutrient broth method approved by clinical and laboratory standard institute (CLSI) were used (Standards, 1993). For these tests, plant extracts in concentrations of 1–10 mg/ml were added to sterilized tube containing nutrient broth and were inoculated with the test microbes. Tubes were incubated using shaker incubator at 37°C for 24 h. MIC was considered that concentration at which no visible bacterial growth was observed.

### Brine shrimp cytotoxicity assay

Cytotoxicity assay was conducted on crude extracts and saponins of *P. hydropiper* against *Artemia salina* (brine-shrimps eggs) following the established procedure (Meyer et al., 1982).

#### Hatching procedure

Sea water is best medium for hatching brine shrimp eggs. Artificial sea water solution was prepared by dissolving commercial salt mixture (38 g) in double distilled water in a shallow rectangular plastic dish of 22 × 32 cm. The plastic dish was divided into two parts using a perforated device. In larger darkened compartment covered with aluminum foil, 50 mg of eggs were sprinkled whereas the smaller compartment was kept open to ordinary light for newly hatched brine shrimps larva. The brine shrimps were incubated for two days at 37°C. After 48 h when the larvae got hatched, it was attracted from dark side using torch and collected using a Pasteur pipette.

#### Samples preparation and application

Plant extracts and crude saponins solutions were prepared in different concentrations ranging from 31.25–1000 μg/ml. Sample solutions corresponding to 31.25, 62.50, 125, 250, 500, and 1000 μg/ml were transferred to separate clean vials and were placed in flow hood to evaporate the solvent. One ml of simulated seawater and 30 shrimps were transferred to each vial and final volume was raised to 5 ml using simulated seawater and pH was adjusted to 7.4. Similar procedure was followed for positive control etoposide and negative control (solvent system) vials. All vials were incubated at 26 ±1°C under illumination for 24 h. The number of survived shrimps were counted in control and test vials and median lethal concentrations (LC_50_) were calculated from dose response curve using Microsoft Excel programme (Zeb et al., 2014).

### MTT cells viability assay

Mouse embryonic fibroblast NIH/3T3 cell line were cultured in DMEM medium supplied with 10% FBS and antibiotics (50 units/ml penicillin and 50 units/ml streptomycin) at 37°C in a humidified atmosphere containing 5% CO_2_. Samples' cytotoxicity against cultured NIH/3T3 cells was determined using MTT assay. NIH/3T3 cells were seeded into 96-well plates at an initial seeding density of 8.0 × 10^3^ cells/well in 200 μl medium followed by incubation for 24 h. Thereafter, the culture medium was removed and replaced with 200 μl medium containing serial dilutions (0.0625–1 mg/ml) of samples. Cells incubated with the media alone were used as positive control and the cells were grown for another 24 h. Subsequently, 20 μl of MTT solution (5 mg/ml) in PBS was added to each well. After incubating the cells for 4 h, the medium containing unreacted dye was removed carefully. The obtained purple formazan crystals were dissolved in 200 μl per well-dimethyl sulfoxide (DMSO) and the absorbance was measured in a micro-plate spectrophotometer reader at a wavelength of 570 nm. The following formula was used to calculate the inhibition of cell growth;

$$\begin{array}{l} \text{Cell viability }\%=\frac{\begin{array}{c} \text{Mean of absorbance value of}\\ \text{treatment group} \end{array}}{\begin{array}{c} \text{Mean of absorbance value of control} \end{array}}\times 100 \end{array}$$

### Estimation of IC_50_ values

Median inhibitory concentrations (IC_50_) for anti-angiogenic assay and median lethal concentrations (LC_50_) were calculated for anti-tumor and cytotoxic activities using Microsoft Excel program.

### Statistical analysis

All the experiments were performed in triplicate and values were expressed as means ± SEM. One-way ANOVA followed by multiple comparison Dunnett's test was used for the comparison of positive control with the test groups. The *P* < 0.05 were considered as statistically significant.

### Cluster analysis

**Figure 1:**
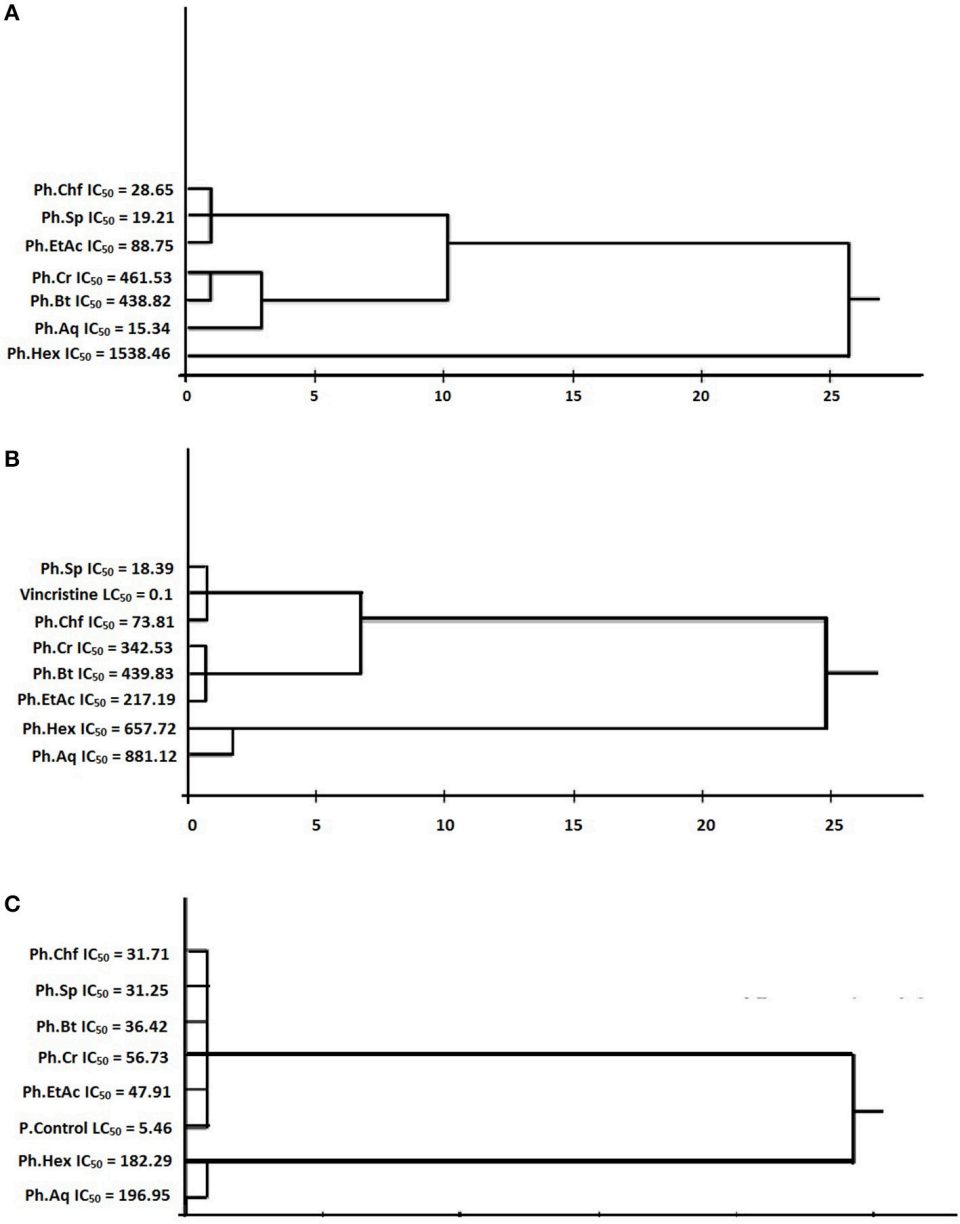
**Cluster analysis based on IC_50_ of various samples. (A)** Cluster analysis and dendogram based on IC_50_ of various samples of *polygonam hydropiper* in CAM assay. **(B)** Cluster analysis and dendogram based on IC_50_ of various samples of *polygonam hydropiper* in anti-tumor assay. **(C)** Cluster analysis and dendogram based on IC_50_ of samples in cytotoxic activity.

Cluster analysis and dendrogram based on IC_50_ and LC_50_ of different samples were developed using SPPS software version 16.0 following Ward's method to draw dendrogram hierarchical clusters. Results are given in Figure 1.

### Regression and linear correlation

**Figure 2:**
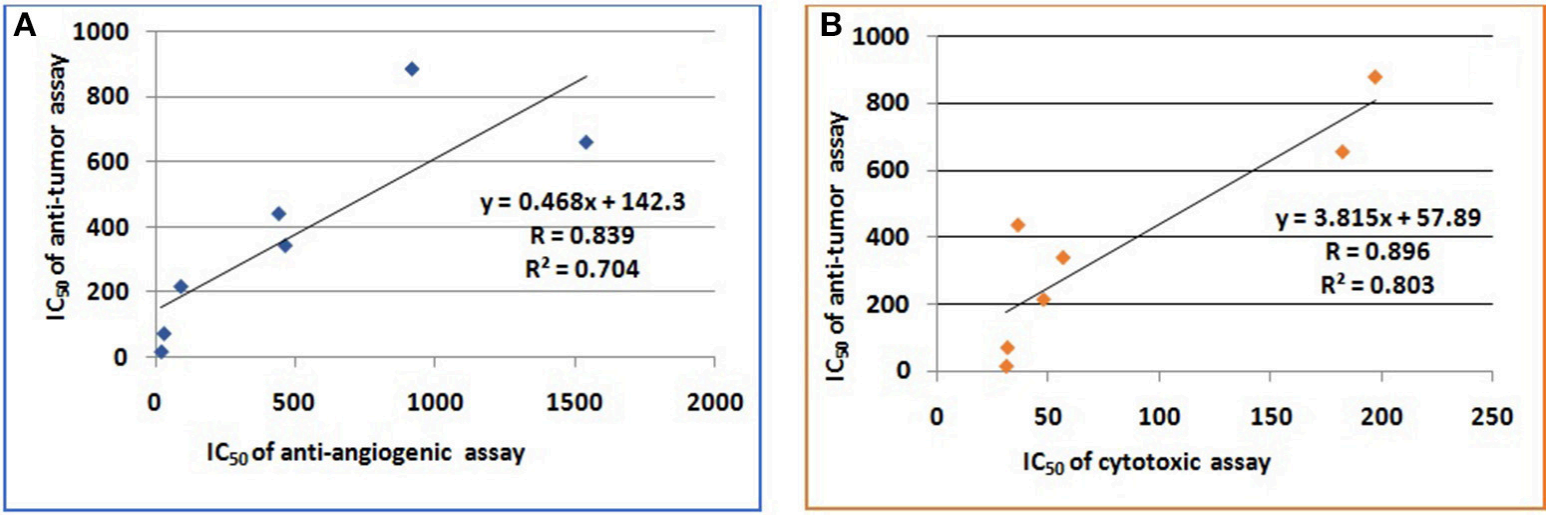
**Linear correlation of IC_50_ of anti-angiogenic-antitumor and IC_50_ of cytotoxic-antitumor activities of *P. hydropiper***. **(A)** Linear correlation of IC_50_ of anti-angiogenic and antitumor activities of *P. hydropiper*. **(B)** Linear correlation of IC_50_ of cytotoxic and antitumor activities of *P. hydropiper*.

Regression (y) and linear correlation (*R*^2^) for anti- angiogenic, anti-tumor, and cytotoxic assays exhibited by Ph.Sp and different fractions were determined using Microsoft Excel 2007. Results are given in Figure 2.

## Results and discussion

Angiogenesis is a rigorously controlled process, regulated by a series of endogenous angiogenic and angiostatic factors under normal circumstances (Folkman and Klagsbrun, 1987). In abnormal angiogenesis like malignancy, atherosclerosis, and chronic inflammations, angiogenesis inhibitors are dominated by angiogenesis promoters leading to abnormal proliferation and migration of the cells. Researchers are looking to isolate and characterize novel angiogenic and anti-angiogenic drugs from natural sources since last fifteen years. In our current study, Ph.Chf, Ph.Sp, Ph.EtAc, and Ph.Cr exhibited highest anti-angiogenic activity causing 78.63 ±1.67, 76.96 ±1.01, 69.43 ±1.15, and 65.33 ±0.88% inhibitions at 1000 μg/ml with IC_50_ of 28.65, 19.21, 88.75, and 461.53 μg/ml, respectively (Table 1). Dexamethasone was used as positive control having IC_50_ value of 11.68 μg/ml. All other fractions showed concentration dependent but less significant activity. In our observations, Ph.Sp showed highest anti-angiogenic activity with IC_50_ of 19.21 μg/ml. Saponins having anti-angiogenic potentials have been isolated previously from plants, like convallamaroside from *Convallaria majalis*, and Polyphyllin D from *Paris polyphylla* (Nartowska et al., 2004; Chan et al., 2011). Likewise, a large number of plants including, crude extracts from *Viscum album, Populus nigra, Chrysobalanus icaco, Cassia garrettiana, Agaricus blazei* were reported for anti-angiogenic potentials. The isolated compounds including, shikonin from *Lithospermum erythrorhizon*, torilin from *Torilis japonica*, Deoxypodophyllotoxin from *Pulsatilla koreana*, resveratrol from grapes, epigallocatechin gallate from green tea, genistein from ginseng and isoliquiritin from licorice have been reported for anti-angiogenic activities both *in-*vitro and *in-*vivo (Sasamura et al., 2004; Wang et al., 2004; Farina et al., 2006).

**Table 1:** **Results of anti-angiogenic assay of *P. hydropiper* extracts and saponins**.

**Samples**	**Percent anti-angiogenic activity Mean ± SEM (***n*** = 5)**						**IC_50_ μg/ml**
	**31.25 μg/ml**	**62.5 μg/ml**	**125 μg/ml**	**250 μg/ml**	**500 μg/ml**	**1000 μg/ml**	
Ph.Cr	29.25 ± 0.20^***^	36.32 ± 3.50^***^	40.50 ± 0.58^***^	45.96 ± 1.01^***^	52.33 ± 0.33^***^	65.33 ± 0.88^***^	461.53
Ph.Hex	21.93 ± 0.49^***^	25.32 ± 1.30^***^	26.66 ± 0.89^***^	32.50 ± 0.86^***^	39.83 ± 1.36^***^	43.53 ± 0.46^***^	1538.46
Ph.Chf	51.00 ± 1.50^***^	55.00 ± 2.80^***^	61.33 ± 0.68^***^	60.93 ± 1.21^***^	69.96 ± 2.66^***^	78.63 ± 1.67^***^	28.65
Ph.EtAc	43.50 ± 0.58^***^	48.50 ± 0.86^***^	52.70 ± 1.60^***^	56.03 ± 0.82^***^	61.00 ± 1.15^***^	69.43 ± 1.15^***^	88.75
Ph.Bt	24.03 ± 0.23^***^	27.00 ± 1.15^***^	28.33 ± 0.33^***^	39.33 ± 0.88^***^	52.66 ± 1.45^***^	61.46 ± 2.43^***^	438.82
Ph.Aq	19.74 ± 0.68^***^	26.61 ± 1.70^***^	31.38 ± 0.68^***^	37.33 ± 0.33^***^	41.00 ± 1.15^***^	52.83 ± 1.36^***^	915.34
Ph.Sp	53.64 ± 1.60^***^	57.22 ± 0.23^***^	59.87 ± 0.26^***^	64.10 ± 1.15^**^	68.43 ± 0.97^*^	76.96 ± 1.01^ns^	19.21

Dexamethasone was used as positive control having IC_50_ value of 11.68 μg/ml.

Values significantly different when compared to standard drug

* p < 0.05,

** p < 0.01, and

*** p < 0.001 at 90% confidence interval.

ns, Values not significantly different in comparison to standard drug.

Cancer is characterized by hysterical and abnormal proliferation of the cells and present more than hundred clinical pathologies (Zeb et al., 2014). The relationship between tumor and tumor-induced angiogenesis has been described by cell adhesion, proteolysis, and migration of cells. There are sound evidences regarding the tumor cells, which have the ability to invade the surrounding tissue and activate the formation of new capillaries from endothelial cell which leads to growth and dissemination of cancer (Sini et al., 2005). So the anti-tumor potential of a specific sample may also correspond to the anti-angiogenic potential of that sample. Similarly, the selection of NIH/3T3 cell line in viability assay is based on the fact that various cell line including the NIH/3T3 mouse embryonic fibroblast, HeLa cell line, chicken embryo fibroblasts, Chinese hamster ovary cells etc. have been reported to be sensitive to sarcoma virus focus formation and leukemia virus propagation and the transfection has been previously evaluated by monitoring immunofluorescence assays (Gorman et al., 1982).

Due to the diverse nature of cancer, the development of cost-effective and broad spectrum cytotoxic drugs is a real challenge to the researchers. Anticancer drugs and radiation causing DNA mutations in actively dividing cells were anticipated to selectively kill cancer cells and produce limited effects on normal cells. Unfortunately, these agents are effective only against certain types of cancer and are associated with toxic effects on normal dividing cell and serious side effects. Therefore, the search for new anticancer drugs both from natural and synthetic origin is crucial. Anti-tumor and brine shrimp lethality bioassays are rapid and economical tools for preliminary cytotoxicity study of plant crude extracts, isolated compounds, and synthetic compounds to develop new anticancer drugs (Meyer et al., 1982; Amara et al., 2008). In anti-tumor assay, Ph.Sp, Ph.Chf, Ph.EtAc, and Ph.Cr showed highest activity causing 90.00 ±1.9 (IC_50_ 18.39 μg/ml), 85.53 ±2.2 (IC_50_ 73.81 μg/ml), 80.00 ±1.9 (IC_50_ 217.19 μg/ml), and 72.20 ±2.2% (IC_50_ 342.53 μg/ml) tumor inhibitions, respectively, at 1000 μg/ml (Table 2). Based on these results Ph.Chf and Ph.EtAc were subjected to activity guided isolation of novel bioactive compounds. In anti-tumor and brine shrimps cytotoxicity assays, Ph.Sp was observed to be highly active fraction. Previously, julibrosides isolated from *Albizia julibrissin* and avicins from *Acacia victoria* were reported as putative anticancer saponins (Lemeshko et al., 2006; Hua et al., 2009).

**Table 2:** **Anti-tumor investigations of extracts and saponins isolated from *Polygonum hydropiper***.

**Samples**	**Concentrations (μg/ml)**	**Average inhibition**	**Percent inhibition**	**LC_50_ (μg/ml)**
Ph.Cr	1000	21.66 ± 0.66	72.20 ± 2.2^***^	342.53
	500	17.66 ± 0.33	58.86 ± 1.1^***^	
	250	13.00 ± 1.15	43.33 ± 3.8^***^	
	125	09.33 ± 0.88	31.10 ± 2.9^***^	
	62.5	09.33 ± 0.33	31.10 ± 1.1^***^	
	31.25	06.00 ± 0.57	20.00 ± 1.9^***^	
Ph.Hex	1000	17.00 ± 0.00	56.66 ± 0.0^***^	657.72
	500	14.33 ± 0.88	47.76 ± 2.9^***^	
	250	11.33 ± 0.33	37.76 ± 1.1^***^	
	125	08.66 ± 0.88	28.86 ± 2.9^***^	
	62.5	07.00 ± 1.73	23.33 ± 5.7^***^	
	31.25	05.33 ± 1.45	17.76 ± 4.8^***^	
Ph.Chf	1000	25.66 ± 0.66	85.53 ± 2.2^*^	73.81
	500	21.33 ± 0.88	71.10 ± 2.9^***^	
	250	17.00 ± 0.57	56.66 ± 1.9^***^	
	125	16.66 ± 0.00	55.53 ± 0.0^***^	
	62.50	14.33 ± 0.88	47.76 ± 2.9^***^	
	31.25	14.33 ± 0.33	47.76 ± 1.1^***^	
Ph.EtAc	1000	24.00 ± 0.57	80.00 ± 1.9^**^	217.19
	500	19.33 ± 0.33	64.43 ± 1.1^***^	
	250	15.66 ± 0.33	52.20 ± 1.1^***^	
	125	13.00 ± 1.15	44.33 ± 3.8^***^	
	62.50	11.66 ± 0.66	38.66 ± 2.2^***^	
	31.25	10.00 ± 0.00	33.33 ± 0.0^***^	
Ph.Bt	1000	20.00 ± 0.57	66.66 ± 1.9^***^	439.83
	500	16.00 ± 0.57	53.33 ± 1.9^***^	
	250	11.33 ± 0.88	37.76 ± 2.9^***^	
	125	10.00 ± 0.57	33.33 ± 1.9^***^	
	62.50	07.66 ± 0.33	25.53 ± 1.1^***^	
	31.25	06.00 ± 0.57	20.00 ± 1.9^***^	
Ph.Aq	1000	16.66 ± 0.66	55.53 ± 2.2^***^	881.12
	500	11.00 ± 0.00	36.66 ± 0.0^***^	
	250	09.00 ± 1.15	30.00 ± 3.8^***^	
	1250	07.00 ± 1.73	23.33 ± 5.7^***^	
	62.5	06.33 ± 1.45	21.10 ± 4.8^***^	
	31.25	04.33 ± 0.88	14.43 ± 2.9^***^	
Ph.Sp	1000	27.00 ± 0.57	90.00 ± 1.9^ns^	18.39
	500	24.00 ± 0.00	80.00 ± 0.0^ns^	
	250	22.33 ± 0.33	74.43 ± 1.1^*^	
	125	21.00 ± 1.15	70.00 ± 3.8^***^	
	62.50	18.00 ± 0.57	60.00 ± 1.9^***^	
	31.25	17.33 ± 0.88	57.76 ± 2.9^***^	

Vincristine sulfate was used as positive control having IC_50_ < 0.1 μg/ml. Values significantly different as compare to positive control,

* P < 0.05,

** P < 0.01,

*** :P < 0.001.

ns, Values not significantly different in comparison to standard drug.

Disc diffusion and MICs methods were used to evaluate the inhibitory effect of test samples against *A. tumefaciens*. Ideally, the sample should not inhibit the growth of *A. tumefaciens* which is responsible for the induction of tumors. As indicated from our results (Table 3), majority of the samples were inactive against *A. tumefaciens* both in disc diffusion and MICs assays. However, Ph.Cr and Ph.Sp showed low antibacterial activity.

**Table 3:** **Results of antibacterial activity (Disc diffusion) and (MICs) against *A. tumefaciens***.

**Samples**	**Diameter of the inhibitory zone (mm) Mean ± SEM (***n*** = 5)**				**MICs (μg/ml)**
	**1.25 mg/ml**	**2.5 mg/ml**	**5 mg/ml**	**10 mg/ml**	**2285.00 ± 7.64**
Ph.Cr	NA	NA	NA	16.66 ± 0.83	NA
Ph.Hex	NA	NA	NA	NA	NA
Ph.Chf	NA	NA	NA	NA	NA
Ph.EtAc	NA	NA	NA	NA	NA
Ph.Bt	NA	NA	NA	NA	NA
Ph.Aq	NA	NA	NA	NA	NA
Ph.Sp	NA	NA	11.00 ± 0.15	13.00 ± 0.50	1523.33 ± 8.81
Ceftriaxone	15.66 ± 0.66	29.33 ± 0.88	24.00 ± 1.00	27.00 ± 0.57	17.50 ± 1.44
Cefotaxime	18.00 ± 0.57	22.00 ± 1.00	2626.33 ± 0.33	31.00 ± 1.52	6.66 ± 1.66

NA, Not active at the tested concentration.

In cytotoxicity assay, Ph.Chf, Ph.EtAc, Ph.Bt, and Ph.Sp showed 100% lethality against the brine shrimps at 1000 μg/ml concentration (Table 4). Dose dependent lethality was observed for all fractions. Ph.Sp, Ph.Chf, Ph.Bt, Ph.EtAc, and Ph.Cr were most potent among the tested samples, exhibiting LD_50_ of 31.25, 31.71, 36.42, 47.91, and 56.73 μg/ml, respectively. Standard drug Etoposide exhibit LD_50_ value of 5.46 μg/ml (Table 4). In MTT cells viability assay, Ph.Chf, Ph.EtAc, and Ph.Sp were most active causing 79.00, 72.50, and 71.50% cytotoxicity, respectively, at 1000 μg/ml. The LC_50_ were 140, 160, 175, 280, and 560 μg/ml for Ph.Chf, Ph.EtAc, Ph.Sp, Ph.Cr, and Ph.Hex, respectively (Table 5). Based on our results, active fractions were subjected to activity guided isolation of novel anticancer agents.

**Table 4:** **Results of brine shrimps cytotoxicity assay performed on *P. hydropiper* extracts and saponins**.

**Samples**	**Conc. (μg/ml)**	**No. of shrimps**	**Average lethality**	**Percent Lethality**	**LC_50_ (μg/ml)**
Ph.Cr	1000	30	28.67 ± 0.33	95.56 ± 1.1^**^	56.73
	500	30	27.00 ± 0.58	90.00 ± 1.9^ns^	
	250	30	24.67 ± 0.88	82.23 ± 2.9^***^	
	125	30	20.33 ± 1.20	67.76 ± 4.0^***^	
	62.50	30	16.00 ± 1.15	53.33 ± 3.8^***^	
	31.25	30	11.33 ± 0.88	37.76 ± 2.9^***^	
Ph.Hex	1000	30	22.00 ± 1.00	73.33 ± 3.3^***^	182.29
	500	30	19.33 ± 0.33	64.43 ± 1.1^***^	
	250	30	16.67 ± 0.67	55.56 ± 2.2^***^	
	125	30	13.00 ± 0.00	43.33 ± 0.0^***^	
	62.50	30	07.33 ± 1.20	34.43 ± 4.0^***^	
	31.25	30	05.67 ± 0.33	18.90 ± 1.1^***^	
Ph.Chf	1000	30	30.00 ± 0.00	100.0 ± 0.0^ns^	31.71
	500	30	27.33 ± 0.88	91.10 ± 2.9^***^	
	250	30	27.67 ± 0.67	92.23 ± 2.2^ns^	
	125	30	25.67 ± 0.33	85.56 ± 1.1^ns^	
	62.50	30	19.00 ± 0.58	63.33 ± 1.9^ns^	
	31.25	30	15.00 ± 1.15	50.00 ± 3.8^ns^	
Ph.EtAc	1000	30	30.00 ± 0.00	100.0 ± 0.0^ns^	47.91
	500	30	28.67 ± 0.88	95.56 ± 2.9^**^	
	250	30	25.33 ± 1.20	84.43 ± 4.0^***^	
	125	30	24.00 ± 1.00	80.00 ± 3.3^**^	
	62.50	30	18.33 ± 1.20	61.00 ± 4.0^*^	
	31.25	30	11.33 ± 0.33	37.76 ± 1.1^***^	
Ph.Bt	1000	30	30.00 ± 0.00	100.0 ± 0.0^ns^	36.42
	500	30	26.67 ± 0.33	88.90 ± 1.1^***^	
	250	30	26.00 ± 1.00	86.66 ± 3.3^***^	
	125	30	21.67 ± 0.33	72.23 ± 1.1^***^	
	62.50	30	17.00 ± 0.00	56.66 ± 0.0^***^	
	31.25	30	14.67 ± 0.67	48.90 ± 2.2^ns^	
Ph.Aq	1000	30	24.33 ± 0.33	81.10 ± 1.1^***^	196.95
	500	30	21.33 ± 1.20	71.10 ± 4.0^***^	
	250	30	16.33 ± 0.88	54.43 ± 2.9^***^	
	125	30	12.67 ± 0.67	42.23 ± 2.2^***^	
	62.50	30	09.67 ± 0.88	32.23 ± 2.9^***^	
	31.25	30	04.33 ± 0.33	14.43 ± 1.1^***^	
Ph.Sp	1000	30	30.00 ± 0.00	100.0 ± 0.0	31.25
	500	30	30.00 ± 0.00	100.0 ± 0.0	
	250	30	28.00 ± 0.58	93.33 ± 1.9	
	125	30	26.33 ± 0.33	87.76 ± 1.1	
	62.50	30	19.33 ± 0.67	64.43 ± 2.2	
	31.25	30	15.00 ± 0.00	50.00 ± 0.0	
Negative control	**—**	30	0	0	—-

Values significantly different as compare to positive control,

* P < 0.05,

** P < 0.01,

*** P < 0.001.

ns, Values not significantly different in comparison to standard drug.

**Table 5:** **Results of cytotoxicty study using mouse embryonic fibroblast NIH/3T3 cell lines**.

**Sample**	**Concentration (μg/mL)**	**Percent cell viability**	**% Cytotoxicity**	**LC_50_ (μg/mL)**
Ph.Cr	1000	30.76 ± 0.50	69.24^***^	280
	500	41.45 ± 0.66	58.55^***^	
	250	53.00 ± 1.00	47.00^***^	
	125	60.00 ± 2.30	40.00^***^	
	62.5	65.00 ± 0.00	35.00^***^	
Ph.Chf	1000	21.00 ± 0.57	79.00^***^	140
	500	26.00 ± 1.15	74.00^***^	
	250	43.00 ± 0.00	57.00^***^	
	125	48.00 ± 0.00	52.00^***^	
	62.5	55.00 ± 0.50	45.00^***^	
Ph.Hex	1000	41.00 ± 0.16	59.00^***^	560
	500	55.00 ± 1.15	45.00^***^	
	250	67.50 ± 0.44	32.50^***^	
	125	72.00 ± 0.00	28.00^***^	
	62.5	78.00 ± 0.00	22.00^***^	
Ph.EtAc	1000	27.50 ± 1.04	72.50^***^	160
	500	37.00 ± 1.15	63.00^***^	
	250	45.00 ± 0.16	55.00^***^	
	125	58.00 ± 0.00	42.00^***^	
	62.5	66.00 ± 0.00	34.00^***^	
Ph.Bt	1000	45.00 ± 0.58	55.00^***^	780
	500	58.33 ± 0.88	41.67^***^	
	250	71.00 ± 1.15	29.00^***^	
	125	79.00 ± 0.57	21.00^***^	
	62.5	84.00 ± 0.00	16.00^***^	
Ph.Sp	1000	28.50 ± 1.00	71.50^***^	175
	500	34.66 ± 1.33	65.34^***^	
	250	46.00 ± 0.00	54.00^***^	
	125	55.00 ± 1.15	45.00^***^	
	62.5	67.00 ± 0.00	33.00^***^	
Ph.Aq	1000	46.66 ± 1.20	53.34^***^	790
	500	50.66 ± 0.88	49.34^***^	
	250	58.00 ± 1.15	42.00^***^	
	125	69.00 ± 0.57	31.00^***^	
	62.5	81.00 ± 0.00	19.00^***^	
Negative control	—–	100	0	—-

Positive control (Standard drug) etoposide, LD_50_ was 5.46 μg/ml.

Values significantly different when compared to standard drug

*** p < 0.001.

In GC, GC–MS analysis of Ph.Cr, Ph.Hex, Ph.Bt, and Ph.EtAc, 126, 124, 131, and 164 compounds were identified, respectively. In Ph.Cr, neophytadiene (100%), 3, 7, 11, 15-Tetramethyl-2-hexadecen-1 (42.52%) and Hexa-hydro-farnesol (32.32%) were most abundant compounds. In GC–MS analysis of Ph.EtAc, coumaran (100%), p-Vinylguaiacol (49.77), and Alpha santolina alcohol (39.26%) were found in higher concentrations. Furthermore, In analysis of Ph.Hex, 9, 12, 15-Octadecatrienoic acid, methyl ester (64.2%), caryophyllene oxide (55.87%), methyl palmitate (55.71%), and drimenol (52.65%) were high concentration compounds. Whereas, methyl linolenate (25.30%) and methyl palmitate (25.32%) were present in high concentrations in Ph.Chf. In Ph.Bt, coumaran (100%), p-Vinylguaiacol (32.71%) and Borneol (26.65%) were find in higher concentrations.

**Figure 3:**
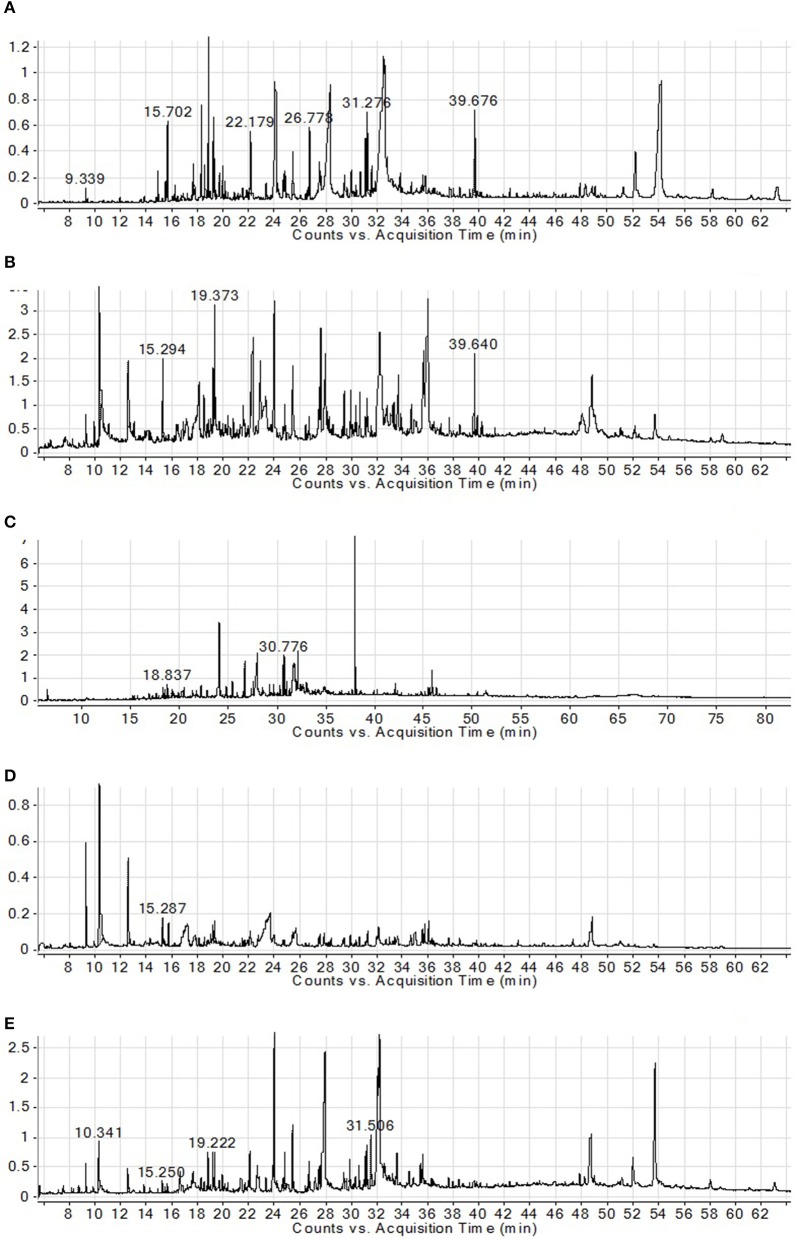
**GC–MS chromatograms of Ph.Cr (A), Ph.Hex (B), Ph.EtAc (C), Ph.Bt (D), and Ph.Chf (E) isolated from *P.hydropiper***.

**Figure 4:**
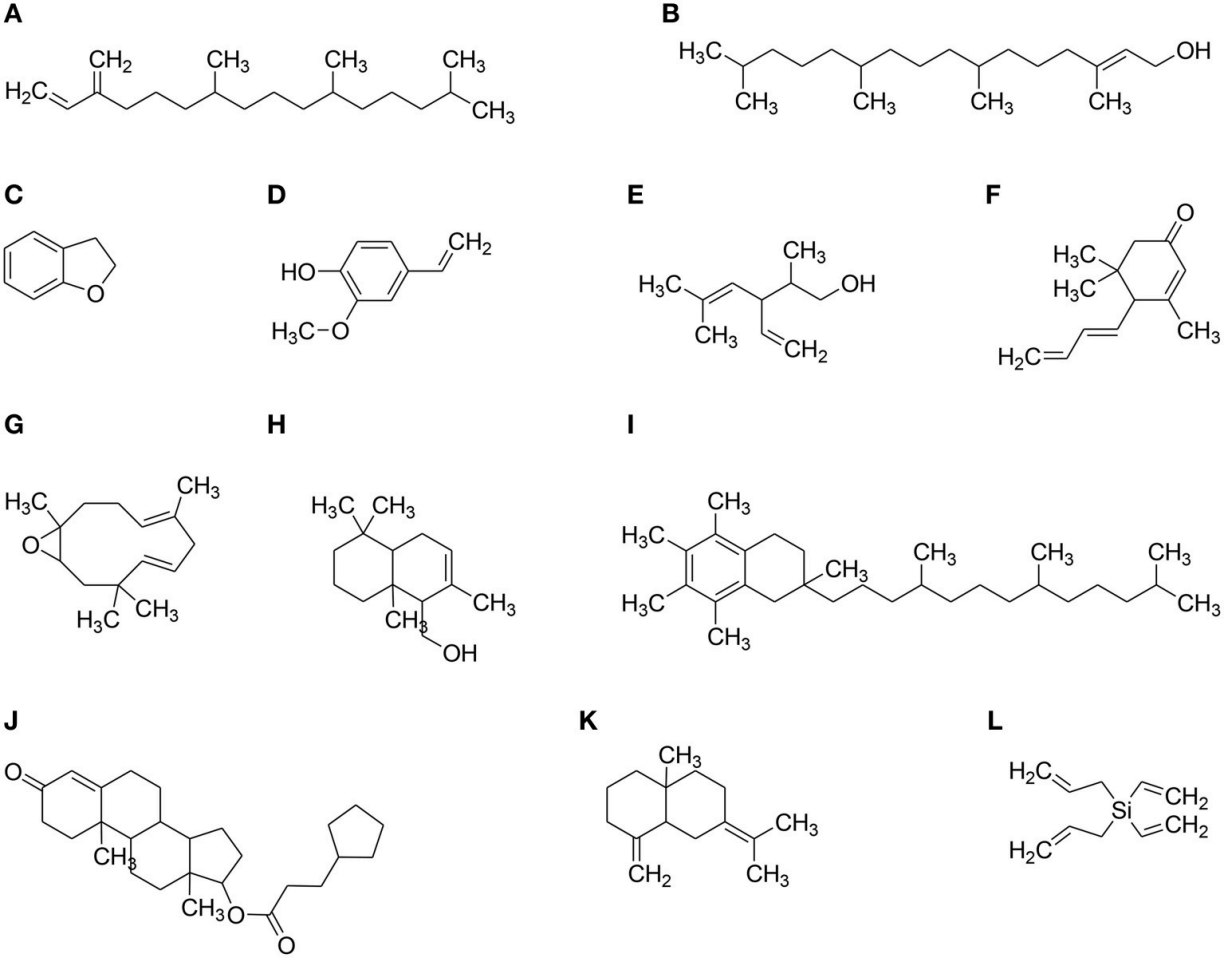
**Major structure identified via GC–MS in different solvent extracts of *P. hydropiper.* (A)** Neophytadiene, **(B)** 7,11,15-Tetramethyl-2-hexadecen-1-ol (Phytol), **(C)** Dihydrobenzofuran, **(D)** p-Vinylguaiacol, **(E)** alpha santolina alcohol, **(F)** Megastigmatrienone, **(G)** Humulene Oxide, **(H)** Driminol, **(I)** alpha Tocopherol, **(J)** Testosterone cypionate, **(K)** Gamma Selinene, and **(L**) Diallyldivinyl silane.

**Figure 5:**
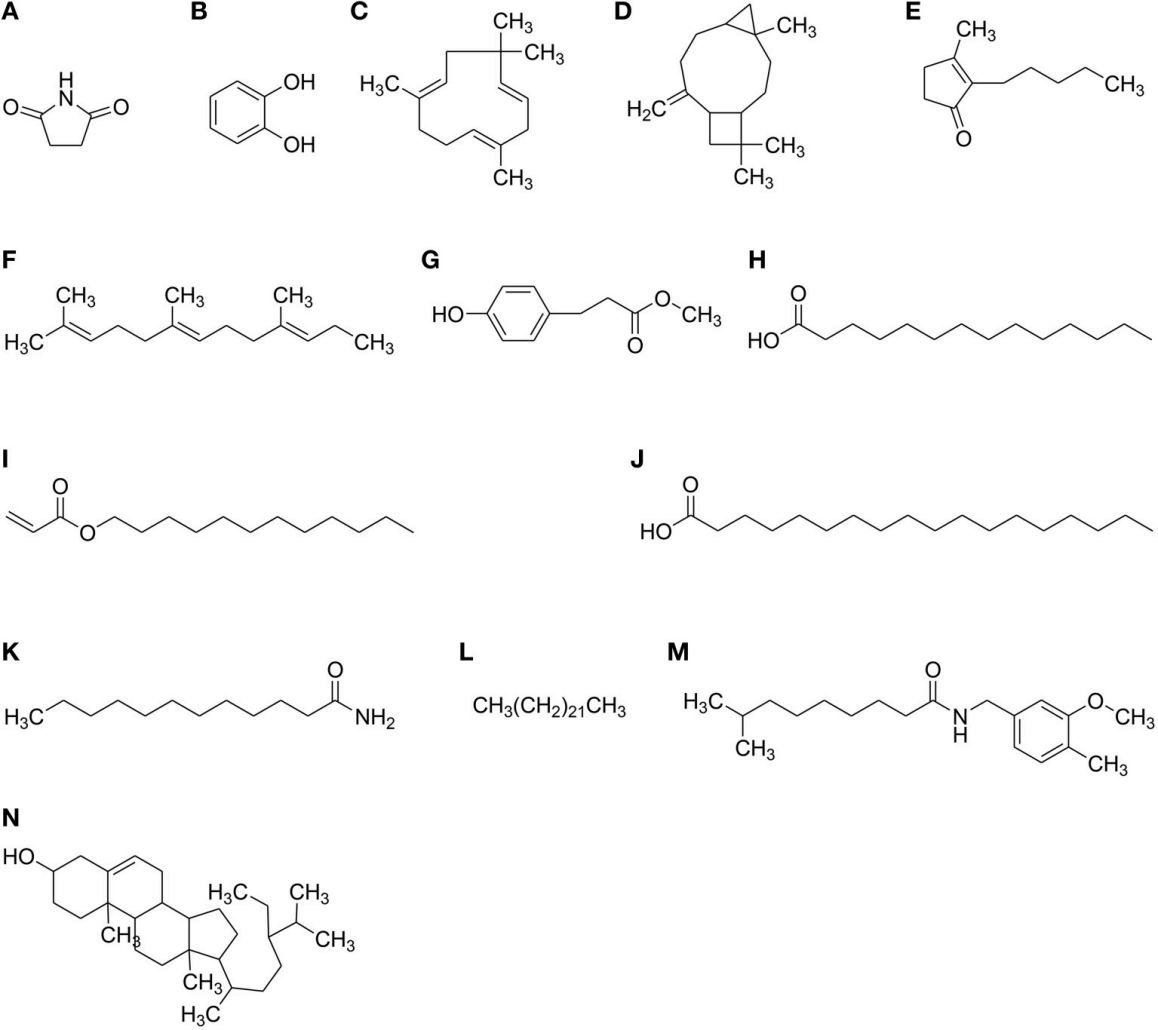
**Structures of active anticancer compounds identified in GC–MS analysis of *Polygonum hydropiper*. (A)** Succinimide **(B)** Pyrocatechol **(C)** Humulene **(D)** Caryophyllene oxide **(E)** Dihydrojasmone **(F)** Farnesol **(G)** Methyl p-coumarate **(H)** Myristic acid **(I)** Dodecyl acrylate **(J)** Stearic acid **(K)** Lauramide **(L)** Tricosane **(M)** Capsaicine **(N)** Clionasterol.

Anticancer compounds have been sorted out in the data obtained from GC–MS analysis of each fraction of *P. hydropiper* i.e., Ph.Cr, Ph.EtAc, and Ph.Bt as shown in Figures 3–5. The GC–MS of Ph.Cr manifested the identification of numerous anticancer compounds as per literature survey namely; dihydrobenzofuran, vinylguaiacol, succinimide, pyrocatechol, humulene, caryophyllene oxide, dihydrojasmone, farnesol, methyl p-coumarate, myristic acid, dodecyl acrylate, stearic acid, lauramide, capsiacine, and tricosane. Similarly, the anticancer compounds sorted out in Ph.EtAc were monomethyl malonate, pyrrolidinone derivatives, succinimide, pyrocatechol, pyrogallol, adamantane, paraben, and dihydrojasmone. Likewise, the anticancer compounds identified in Ph.Bt were succinimide, pyrocatechol, borneol, benzeneacetic acid, pyrogallol, nicotinamide, caryophyllene oxide, atlantone, and palmitic acid. Some of the compounds were found in all the fractions, for instance succinimide and pyrocatechol, which have been reported as strong anticancer agents (Hall et al., 1995; Taysse et al., 1995). Putatively known anticancer compounds i.e., Dihydrojasmone, capsaicine and caryophyllene oxide have also been identified in the chromatograms of our sample (Richeux et al., 1999; Flescher, 2005; Jun et al., 2011). Fatty acids and their derivative having anticancer potential have also been found in these samples, for instance myristic acid, dodecyl acrylate, stearic acid, lauramide, and palmitic acid (Fermor et al., 1992; Yoshii, 1997; Harada et al., 2001; Li et al., 2011). Similarly, dihydrobenzofuran has also been revealed with significant anticancer potentials and its derivatization is still in progress by several researchers (Choi et al., 2015). Likewise, vinylguaiacol is considered as a potential antioxidant compound (Azadfar et al., 2015). In the same way, the Humulene has also been reported to inhibit tumor growth (Legault et al., 2003). The GC–MS also indicates the presence of tocopherol, which is a well-known antioxidant and anticancer vitamin (Yu et al., 2009). Likewise, Silane derivatives has been demonstrated as an effective agent in a nanoparticle based drug delivery system for anticancer compounds (He et al., 2010).

## Conclusions

Results of the current study indicate that *P. hydropiper* possesses broad spectrum cytotoxic activities. Samples were inactive against *A. tumefaciens in-vitro*, which indicate that this is a useful anti-tumor assay for *P. hydropiper.* GC–FID and GC–MS analysis revealed the presence of a large number of anticancer compounds which may be responsible for the overall anticancer activity of the extracts. Furthermore, studies regarding isolation and purification of novel anticancer components can depict the precise potentials of the plant for the chemotherapy of a variety of cancers. Our findings regarding cytotoxic potentials of extracts and saponins may offer scientific justification for the ethnomedicinal uses of the plant.

## Author contributions

MA and SA carried out experimental work, data collection and evaluation, literature search, and manuscript preparation. MJ and FU supervised research work and helped in study design. MI conducted GC–MS. FS, AS, MK, WA, and GA refined the manuscript for publication. All authors read and approved the final manuscript for publication.

## Funding

This research received no specific grant from any funding agency in the public, commercial, or not for-profit sectors.

### Conflict of interest statement

The authors declare that the research was conducted in the absence of any commercial or financial relationships that could be construed as a potential conflict of interest.
